# Regulation of *PPARGC1A* gene expression in trained and untrained human skeletal muscle

**DOI:** 10.14814/phy2.13543

**Published:** 2017-12-12

**Authors:** Daniil V. Popov, Evgeny A. Lysenko, Pavel A. Makhnovskii, Nadia S. Kurochkina, Olga L. Vinogradova

**Affiliations:** ^1^ Laboratory of exercise physiology Institute of Biomedical problems of the Russian Academy of Sciences Moscow Russia; ^2^ Faculty of Fundamental Medicine M.V. Lomonosov Moscow State University Moscow Russia; ^3^ Department of Genetics Faculty of Biology M.V. Lomonosov Moscow State University Moscow Russia

**Keywords:** Alternative promoter, *PPARGC1A*, skeletal muscle, training level

## Abstract

Promoter‐specific expression of the *PPARGC1A* gene in untrained and trained human skeletal muscle was investigated. Ten untrained males performed a one‐legged knee extension exercise (for 60 min) with the same relative intensity both before and after 8 weeks of cycling training. Samples from the *m. vastus lateralis* of each leg were taken before and after exercise. Postexercise *PPARGC1A* gene expression via the canonical promoter increased by ~100% (*P* < 0.05) in exercised and nonexercised untrained muscles, but did not change in either leg after training program. In untrained and trained exercised muscle, *PPARGC1A* gene expression via the alternative promoter increased by two orders of magnitude (*P* < 0.01). We found increases in postexercise content of dephosphorylated (activated) CRTC2, a coactivator of CREB1, in untrained exercised muscle and in expression of CREB1‐related genes in untrained and trained exercised muscle (*P* < 0.01–0.05); this may partially explain the increased expression of *PPARGC1A* via the alternative promoter. In addition, comparison of the regulatory regions of both promoters revealed unique conserved motifs in the alternative promoter that were associated with transcriptional repressors SNAI1 and HIC1. In conclusion, in untrained muscle, exercise‐induced expression of the *PPARGC1A* gene via the canonical promoter may be regulated by systemic factors, while in trained muscle the canonical promoter shows constitutive expression at rest and after exercise. Exercise‐induced expression of *PPARGC1A* via the alternative promoter relates to intramuscular factors and associates with activation of CRTC2‐CREB1. Apparently, expression via the alternative promoter is regulated by other transcription factors, particularly repressors.

## Introduction

In skeletal muscle, peroxisome proliferator‐activated receptor *γ* coactivator 1*α* (PGC‐1*α*, encoded by the *PPARGC1A* gene) plays an important role in regulating mitochondrial biogenesis and in adaptation to aerobic training. Acute exercise activates PGC‐1*α*, thereby modulating the transcriptional activity of its partners and regulating expression of genes involved in mitochondrial biogenesis, angiogenesis, and fat and carbohydrate metabolism (Scarpulla 2008; Lira et al. 2010; Narkar et al. 2011; Kupr and Handschin 2015). Moreover, an increase in *PPARGC1A* gene expression and PGC‐1*α* protein content occurs several hours after muscle activity, which may stimulate mitochondrial biogenesis at the later stages of recovery (Wright et al. 2007; Ikeda et al. 2008).

More than 7000 human genes have a putative alternative promoter and first alternative exon(s) (Carninci et al. 2006; Kimura et al. 2006). Alternative promoters have the potential to play important roles in generating the complexity of human gene expression, in particular, in regulating gene expression via different promoter in a stimuli‐specific manner. In rodent and human skeletal muscle, the *PPARGC1A* gene can be expressed via the canonical (proximal) and alternative (distal) promoters (Martinez‐Redondo et al. 2015; Popov et al. 2015a). At rest, expression via the canonical promoter is high, while that via the alternative promoter is very low. In rodent (Tadaishi et al. 2011b; Wen et al. 2014) and human (Popov et al. 2015b) muscle, acute exercise of different intensity has a slight effect on expression via the canonical promoter, that is, this promoter has constitutive intensity‐independent expression, but substantially increases expression via the alternative promoter in an intensity‐dependent manner. Thus, *PPARGC1A* gene expression via the alternative promoter makes a greater contribution to exercise‐induced increases in total *PPARGC1A* mRNA than that via the canonical promoter (Popov et al. 2015b). RNA‐seq and exon‐exon junction analysis revealed that, in human skeletal muscle after aerobic exercise, the alternative promoter produces two major isoforms (full‐length *PGC‐1α‐b* and N‐truncated *NT‐PGC‐1α‐b* [or *PGC‐1α4*]) only (Popov et al. 2015b). Expression of other isoforms is negligible. The postexercise level of *PGC‐1α‐b* mRNA is substantially higher than that of all *NT‐PGC‐1α* isoforms, which were evaluated using primers to exons 5 and 7a by qPCR (Ydfors et al. 2013; Popov et al. 2014; Silvennoinen et al. 2015; Conceicao et al. 2016) and RNA‐seq (Popov et al. 2015b). Therefore, in human skeletal muscle after aerobic exercise, the alternative promoter expresses mainly full‐length *PGC‐1α‐b* isoform; this isoform plays an important role in regulation of mitochondrial biogenesis like the full‐length *PGC‐1α‐a* (or *PGC‐1α1*) isoform originating from the canonical promoter (Miura et al. 2008; Tadaishi et al. 2011a; Hatazawa et al. 2015).

The molecular mechanism that regulates expression via the alternative promoter is not clear. In human skeletal muscle, expression of *PPARGC1A* via the alternative promoter depends on exercise‐induced metabolic stress (Norrbom et al. 2011). Studies in human myoblasts (Norrbom et al. 2011) and mice (Wen et al. 2014) show a role for 5’ AMP‐activated protein kinase (AMPK) in regulation of expression via the alternative promoter; however, other studies in myoblasts (Yoshioka et al. 2009), isolated rat muscle (Tadaishi et al. 2011b), and humans (Popov et al. 2017) did not confirm this. Several myoblast and rodent studies clearly show that cAMP‐responsive element‐binding protein 1 (CREB1) regulates expression via the alternative promoter through the *β*2‐adrenoreceptor (AR)‐cAMP‐protein kinase A (PKA) pathway (Chinsomboon et al. 2009; Yoshioka et al. 2009; Tadaishi et al. 2011b; Bruno et al. 2014; Wen et al. 2014). However, studies in humans did not confirm a role for the *β*2AR‐cAMP‐PKA pathway in regulating *PPARGC1A* expression via the alternative promoter (Popov et al. 2015b), or in regulating expression of total *PPARGC1A* mRNA after acute exercise (Brandt et al. 2016) and at rest (Robinson et al. 2010).

These discrepancies might be related to different experimental models (myoblasts, rodents, and human skeletal muscle) and/or to adaptation of skeletal muscle to regular aerobic exercise. Therefore, we investigated promoter‐specific regulation of *PPARGC1A* gene expression in untrained and trained human skeletal muscle. For this purpose, subjects performed one‐legged knee extensions (for 60 min) with the same relative intensity before and after 8 weeks of cycling training. Samples of *m. vastus lateralis* were taken from the exercised and nonexercised legs prior to and after the one‐legged knee extension exercise. This approach allowed us to evaluate the effects of systemic and intramuscular factors on regulation of *PPARGC1A* gene expression via both promoters in untrained and endurance‐trained muscle.

## Methods

### Ethical approval

The study was approved by the Ethics Committee of the Institute and complied with the guidelines set forth in the Declaration of Helsinki. All participants provided written informed consent to participate in the study.

### Design of the study

Ten untrained males (median age, 22 years [interquartile range, 21–26 years]; weight, 74 kg [72–79 kg]; body mass index, 23 kg/m^2^ [22–25 kg/m^2^]) were enrolled in the study. Each participant performed a one‐legged continuous knee extension exercise with the same relative intensity in untrained and trained state (before and after the 8 week aerobic cycling training program, respectively). Biopsies from the *m. vastus lateralis* of both legs were taken prior to and at 2 min, 1 h, and 4 h after each one‐legged continuous knee extension exercise. Intensity of the one‐legged continuous knee extension exercise was selected using an incremental one‐legged ramp test; the ramp test was performed for 48 h before each one‐legged continuous knee extension exercise.

### One‐legged knee extension ramp test

During the first visit to the laboratory, the participants familiarized themselves with the test procedures and completed an incremental one‐legged ramp test on a modified electromagnetic ergometer (Ergometric 900S, Ergoline, Germany) until exhaustion, as described previously (Kuznetsov et al. 2015). The initial load, load increment and knee extension rate were 0 W, 2.5 W/min, and 60 cycles/min, respectively. Three days later, each participant performed an incremental one‐legged ramp test until exhaustion to evaluate the anaerobic threshold (AT) of the *m. vastus lateralis* according to electromyography activity and changes in deoxyhemoglobin content, as described previously (Kuznetsov et al. 2015). EMG activity in the middle part of the *m. vastus lateralis* was measured continuously using a CP511 amplifier (Grass Telefactor, USA) and Ag/AgCl electrodes. Changes at the concentration of deoxyhemoglobin were measured continuously using a tissue infrared spectrometer NIRO‐200 (Hamamatsu Photonics K.K., Japan). The infrared source and receiver were placed 40 mm apart and close to the EMG electrodes; they were fixed onto the skin with tape and an elastic bandage. The incremental one‐legged ramp test until exhaustion was repeated 48 h after the last training session of the 8 week aerobic cycle training program.

### One‐legged continuous knee extension exercise

Participants were instructed not to undertake any exercise for 48 h after the incremental one‐legged ramp test. Then they arrived at the laboratory at 07:00 and ate a standardized breakfast (3582 kJ; 22 g protein, 154 g carbohydrate, 16 g lipids). At 09:15, after a 30 min rest in the supine position, a baseline venous blood sample was taken, along with samples from the *m. vastus lateralis* in each leg (see below). At 09:35, the participants started the one‐legged continuous knee extension exercise (5 min warm up [60% of AT], followed by 55 min at 75% AT). The participants then ate a standardized lunch (3714 kJ; 45 g protein, 183 g carbohydrate, 27 g lipids) 1 h 20 min after the exercise session.

Venous blood was drawn through a catheter placed in the median cubital vein; samples were taken at rest and at 55 min after beginning the exercise. Plasma concentrations of catecholamines were measured using a Catecholamines in Plasma HPLC kit and a high‐performance liquid chromatography (Chromsystems Instruments & Chemicals, Germany). Biopsies from the *m. vastus lateralis* of both legs were taken prior to and at 2 min, 1 h, and 4 h after exercise, under local anesthesia (2 mL 2% lidocaine), using a microbiopsy technique (Hayot et al. 2005). The muscle samples were quickly blotted with gauze to remove superficial blood, frozen in liquid nitrogen, and stored at −80°C until required. The first biopsy was taken 15 cm proximal to the lateral epicondyle of the femur, with each subsequent biopsy taken 2 cm proximal to the previous biopsy. The one‐legged continuous knee extension exercise was repeated 48 h after the second incremental one‐legged ramp test (after the 8 week cycling training program).

### Cycling training program

All individuals participated in the 8 week cycling training program (five sessions/week) using electromagnetic ergometers (Ergoselect 200, Ergoline, Germany). Every second week during the training period, a submaximal incremental cycling test was performed to evaluate power at a blood lactate concentration of 4 mmol/l (LT_4_) and to correct the training load. The training program comprised continuous (60 min, 70% LT_4_) and intermittent ([3 min, 50% LT_4_ + 2 min, 85% LT_4_] × 12) exercises (continuous to intermittent exercise ratio 18:22).

### Measurement of mitochondrial respiration

Mitochondrial respiration was measured in the basal state before and after the 8 week cycling training. A piece of fresh biopsy sample was placed immediately in ice‐cold relaxing buffer BIOPS (2.77 mmol/L CaK_2_EGTA, 7.23 mmol/L K_2_EGTA, 5.77 mmol/L Na_2_ATP, 6.56 mmol/L MgCl_2_·6 H_2_O, 20 mmol/L taurine, 20 mmol/L imidazole, 0.5 mmol/L dithiothreitol, 50 mmol/L MES hydrate, and 15 mmol/L Na‐phosphocreatine), and fiber bundles were separated using a pair of needles. Then, the fibers were incubated for 30 min in 2 mL BIOPS buffer containing saponin (50* μ*g/mL). The fibers were washed twice (10 min each time) in ice‐cold MiR05 buffer (110 mmol L^−1^ sucrose, 60 mmol L^−1^ potassium lactobionate, 0.5 mmol L^−1^ EGTA, 3 mmol L^−1^ MgCl_2_·6H_2_O, 20 mmol L^−1^ taurine, 10 mmol L^−1^ KH_2_PO_4_, 20 mmol L^−1^ HEPES, 1 g L^−1^ BSA, pH 7.1). The mass‐specific oxygen consumption rate of ~1.5 mg muscle fibers (wet weight) in 2 ml buffer MiR05 was then measured at 37°C using an Oxygraph Plus System (Hansatech, UK). To avoid potential oxygen limitation, all experiments were carried out under hyperoxygenated conditions (*C*o_2_ > 180 *μ*mol/L). State 2 respiration (absence of adenylates) was assessed by adding malate (2 mmol/L), pyruvate (5 mmol/L), and glutamate (10 mmol/L). Maximal coupled respiration with convergent electron input to complex I and II of the electron transport system (State 3) was achieved by adding ADP (5 mmol/L) in MgCl_2_ (0.6 mol/L per 1 mol/L ADP), followed by addition of succinate (10 mmol/L). The integrity of the outer mitochondrial membrane was tested by adding cytochrome *c* (10 *μ*mol/L). If respiration remained stable, the quality of the mitochondria was considered sufficient. Next, oligomycin (2 *μ*g/mL) was added to block complex V (leak state). Finally, an uncoupler (trifluoromethoxy carbonylcyanide phenylhydrazone; FCCP) was titrated from 0.25 *μ*mol/L to 1.25 *μ*mol/L.

### Western blot analysis

Frozen tissue samples (~10 mg) were homogenized in ice‐cold RIPA buffer (25 mmol/L Tris‐HCl (pH 7.6), 150 mmol/L NaCl, 1% NP‐40, 1% sodium deoxycholate, 0.1% SDS) containing phosphatase and protease inhibitors (50 mmol/L *β*‐glycerophosphate, 50 mmol/L NaF, 1 mmol/L Na_3_VO_4_, 20 mg/L aprotinin, 50 mg/L leupeptin, 20 mg/L pepstatin, and 1 mmol/L PMSF). The samples were centrifuged for 10 min at 10,000*g* at 4°C, and the protein concentration in the supernatants was measured in a bicinchoninic acid assay. Samples were mixed with Laemmli buffer and loaded onto 7.5–10% polyacrylamide gels (20 *μ*g protein/lane). Electrophoresis (20 mA per gel) was performed using a Mini‐PROTEAN Tetra Cell system (Bio‐Rad, USA). The proteins were transferred to nitrocellulose membranes using a Trans‐Blot Turbo system (Bio‐Rad, USA) in Towbin buffer for 30 min at 25 V. The membranes were stained with Ponceau S to verify consistent loading of proteins, followed by washing and incubation in 5% nonfat dry milk for 1 h. The membranes were subsequently incubated at 4°C overnight with antibodies specific for phospho‐acetyl‐CoA carboxylase (ACC^Ser79/222^; 1:1000; ab68191; Abcam, UK), phospho‐mitogen‐activated protein kinase (p38^Thr180/Tyr182^; 1:500; ab195049; Abcam), phospho‐Ca^2+^/calmodulin‐dependent protein kinase II (CaMKII^Thr286^; 1:2500; ab32678; Abcam), phospho‐CREB1^Ser133^ (1:500; ab32096; Abcam), CREB‐regulated transcription coactivator 2 (CRTC2; 1:500, #13017; Cell Signaling), and oxidative phosphorylation proteins (OXPHOS): NDUFB8, SDHB, UQCRC2, MT‐CO1, and ATP5A1 (1:2500; ab110413; Abcam) followed by incubation for 1 h at room temperature with an anti‐rabbit secondary antibody (Cell Signaling). After each step, the membranes were washed three times for 5 min with PBS‐Tween 20. Finally, the membranes were incubated with ECL substrate (Bio‐Rad, USA) or with SuperSignal West Femto Maximum Sensitivity Substrate (Thermo Scientific, USA), and luminescent signals were captured using a ChemiDoc Imaging System (Bio‐Rad, USA). All data are expressed as the ratio of target protein to the loading control, as evaluated by Coomassie blue staining.

**Figure 1:**
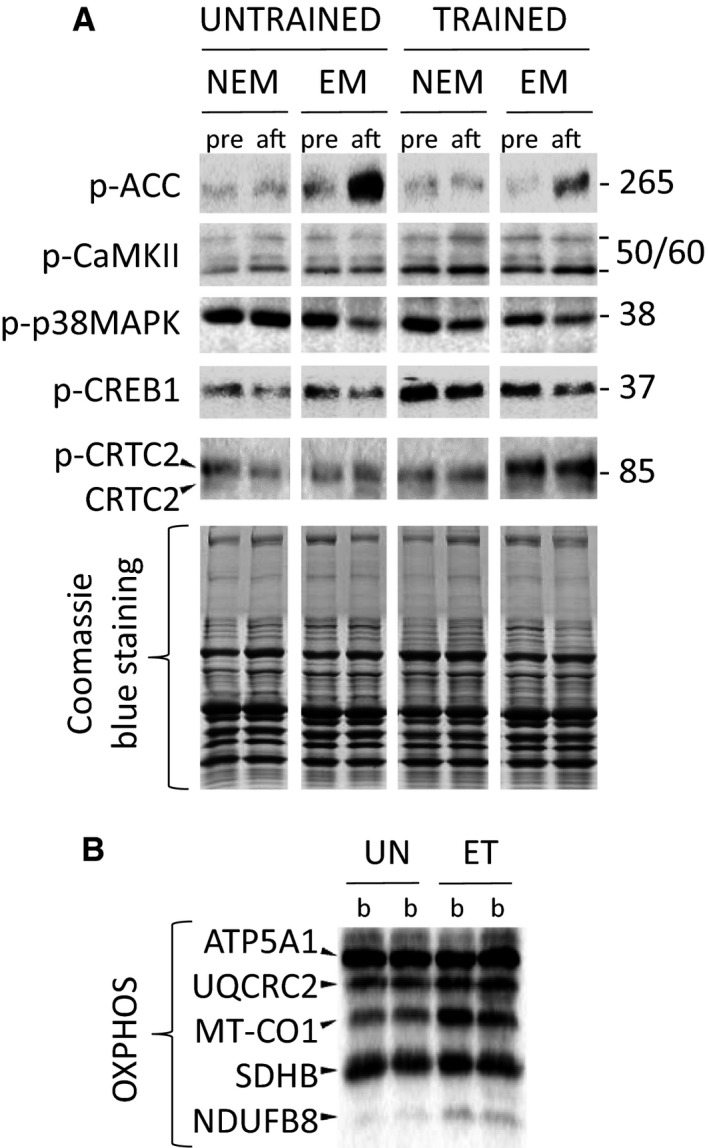
Representative immunoblots. Level of proteins in nonexercised (NEM) and exercised (EM) muscle pre (*pre*), at 2 min after (*aft*) the one‐legged continuous knee extension exercise both before (*untrained*) and after (*trained*) the 8 week endurance training program (A). The basal level (*b*) of OXPHOS proteins in untrained (*UN*) and endurance trained (*ET*) muscle in both legs (B).

CRTC2 migrated within 7.5% polyacrylamide gels as an 85 kDa doublet. Several studies (Screaton et al. 2004; Shaw et al. 2005; Liu et al. 2010) using different experimental approaches (site‐directed mutagenesis, phospho‐specific antibody, and MS analysis) showed the correlation between the mobility‐shifted CRTC2 and phosphorylation at Ser^171^, a principal phosphorylation site on CRTC2. According to this finding, dephosphorylated (activated) CRTC2 was evaluated as the faster‐migrating band (Fig. 1) .

### RNA extraction

Frozen samples (~20 mg) were cut into 20 *μ*m sections using an ultratome (Leica Microsystems, Germany). RNA was extracted using an RNeasy Mini Kit (Qiagen, Germany). RNA concentration and purity were measured using a NanoDrop 2000 spectrophotometer (Thermo Scientific, USA). All RNA samples had an A_260_/A_280_ ratio of 1.95–2.05. After DNase treatment (Thermo Scientific, USA), cDNA was generated from 1 *μ*g aliquots of total RNA using a MMLV reverse transcriptase kit (Evrogen, Russia).

### Real‐time polymerase chain reaction

Real‐time polymerase chain reaction was performed using a Rotor‐Gene Q cycler (Qiagen, Germany). The annealing temperature was optimized for each primer. The thermal profile included an initial denaturation at 95°C for 5 min, followed by 45 cycles of denaturation at 95°C for 15 sec, annealing (56–60°C) for 30 sec, and extension at 72°C for 45 sec. Amplified genes were quantified by measuring fluorescence using the EvaGreen Master Mix (Syntol, Russia). The specificity of the amplification was monitored by analysis of melting curves and by agarose gel (1%) electrophoresis. Each sample was run in triplicate, and a nontemplate control was included in each run. Expression of target gene mRNA was calculated using the efficiency‐corrected ∆Ct method: ${}^{2}\sqrt{{\left(1+E_{\mathrm{ref}1}\right)}^{Ct_{\mathrm{ref}1}}\times {\left(1+E_{\mathrm{ref}2}\right)}^{Ct_{\mathrm{ref}2}}/{\left(1+E_{\mathrm{tar}}\right)}^{Ct_{\mathrm{tar}}}}$. PCR efficiency (*E*) was calculated using standard curves corresponding to the target genes and two reference genes (*RPLP0* and *GAPDH*). Expression of both reference genes remained unchanged under all experimental conditions. Expression of *PPARGC1A* via the canonical and alternative promoters was evaluated using primers complementary to sequences in exons 1a and 2, and in exons 1b and 2 (exon 2 is a common exon for all isoforms), respectively, as previously described (Popov et al. 2017). The primers to exons 1a and 2 detect all transcripts originated from exon 1a (*PGC‐1α‐a* [or *PGC‐1α1*] and *NT‐PGC‐1α‐a*); the primers to exons 1b and 2 detect all transcripts originated from exon 1b (*PGC‐1α‐b*,* PGC‐1α2*, and *NT‐PGC‐1α‐b* [or *PGC‐1α4*]). In human skeletal muscle, the postexercise expression of *PPARGC1A* gene is related mainly to expression of two full‐length isoforms, namely *PGC‐1α‐a* and *PGC‐1α‐b* (Ydfors et al. 2013; Popov et al. 2014, 2015b; Silvennoinen et al. 2015; Conceicao et al. 2016).

### Unique conserved motifs in the alternative promoter of the PPARGC1A gene

We identified putative regulatory regions (−973–+100) in the human alternative promoter, in which open chromatin has been reported, by the Ensembl Regulatory Build (Zerbino et al. 2015). Next, a slightly overlapping region (−1500–+500) in the alternative and the canonical promoters of human and mouse *PPARGC1A* were aligned, and conserved regulatory motifs and corresponding transcription factors were identified by HOCOMOCO (Kulakovskiy et al. 2016). Finally, conserved regulatory motifs and corresponding transcription factors unique to each promoter were chosen.

### Statistical analysis

Because the sample size was small (*n* = 10) and the data were not normally distributed, all data are expressed as the median and interquartile range. The Wilcoxon matched‐pairs signed‐rank test was used to compare measurements; Holm‐Bonferroni correction was applied for the repeated measurements. The level of significance was set at 0.05.

## Results

### Effect of 8 week training

**Figure 2:**
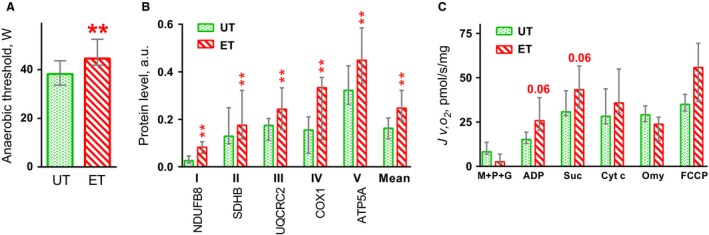
An 8 week cycling training program increased power at the anaerobic threshold in the one‐legged incremental test (A), levels of protein in mitochondrial complexes I–V (B), and ADP‐stimulated respiration in permeabilized muscle fibers (C) in the *m. vastus lateralis* in the basal state. ** *P* < 0.01, endurance‐trained (ET) compared with untrained (UT); *n* = 10 for A and B, *n* = 6 for C. Each value represents the median and interquartile range. Malate (M), pyruvate (P), glutamate (G), succinate (Suc), cytochrome *c* (Cyt *c*), oligomycin (Omy), trifluoromethoxy carbonylcyanide phenylhydrazone (FCCP).

Cycling training increased AT in the one‐legged incremental test by 17% (*P* < 0.01) and the basal level of mitochondrial proteins in complexes I–V by ~35–215% (*P* < 0.01); a tendency to increased ADP‐stimulated respiration rate of mitochondria in permeabilized muscle fibers by ~50% (*P* = 0.06, *n* = 6) in the basal state was found (Fig. 2). We found the increase in the basal phosphorylation of CaMKII^Thr286^ (*P* < 0.01) and CREB1^Ser133^ (*P* < 0.05) in endurance‐trained muscle compared to untrained muscle (Fig. 6).

### Effect of acute one‐legged continuous knee extension exercise on physiological indices

**Figure 3:**
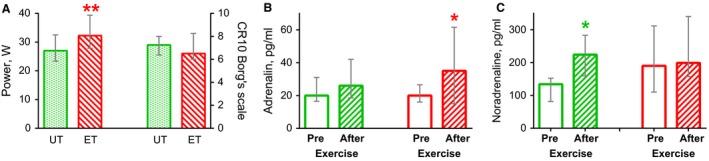
Intensity of one‐legged continuous knee extension exercise, mean subjective rating of exertion (A) and blood level of catecholamines (B and C) in untrained state (*UT*, green bars) and after the 8 week endurance training (*ET*, red bars). * *P* < 0.05 and ** *P* < 0.01, compared with untrained muscle; *n* = 10 for A, *n* = 9 for B and C. Each value represents the median and interquartile range.

Mean power in the one‐legged continuous knee extension exercise increased by 18% (8–12%; *P* < 0.01) after cycling training. However, physiological responses to the one‐legged knee extension exercise, evaluated by subjective rating of perceived exertion and increased blood catecholamine levels, were similar before and after the 8 week training program (Fig. 3).

### Effect of acute one‐legged continuous knee extension exercise on gene expression

**Figure 4:**
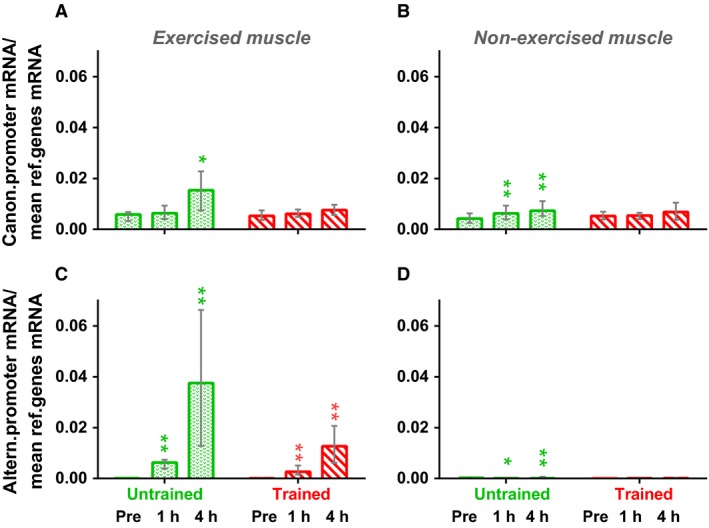
Expression of the *PPARGC1A* gene in the exercised and nonexercised *m. vastus lateralis* via the canonical (A and B) and alternative (C and D) promoters at 1 h, and 4 h after one‐legged continuous knee extension exercise both before (green bars) and after (red bars) the 8 week cycling training program. * *P* < 0.05 and ** *P* < 0.01, compared with untrained muscle; *n* = 9–10. Each value represents the median and interquartile range.

In the untrained state, one‐legged knee extension exercise increased expression of *PPARGC1A* via the canonical promoter in exercised (*P* < 0.05) and nonexercised (*P* < 0.01) muscle by ~100%; however, no changes were observed in either leg after the training program. Postexercise expression of *PPARGC1A* via the alternative promoter in untrained (*P* < 0.01) and trained (*P* < 0.01) exercised muscles increased by two orders of magnitude. One‐legged knee extension exercise increased expression via the alternative promoter (*P* < 0.01) in nonexercised muscle before training, but the postexercise mRNA levels were very low and most likely played no role from physiological point of view; trained nonexercised muscle showed no changes in expression via the alternative promoter (Fig. 4).

**Figure 5:**
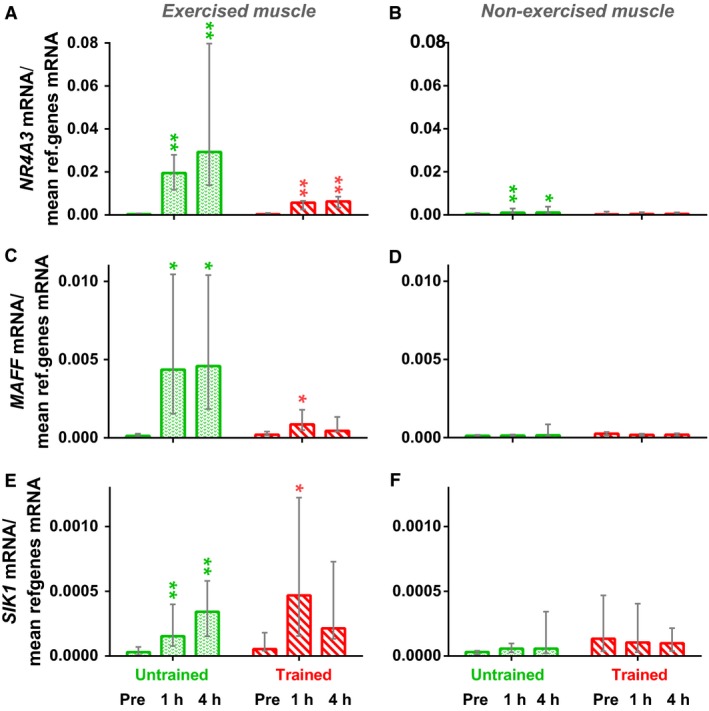
Expression of CREB1‐related genes (*NR4A3*,*MAFF*, and *SIK1*) in the exercised (A, C, and E) and nonexercised (B, D, and F) *m. vastus lateralis* at 1 h, and 4 h after the one‐legged continuous knee extension exercise both before (green bars) and after (red bars) the 8 week cycling training program. * *P* < 0.05 and ** *P* < 0.01 compared with untrained muscle; *n* = 9–10. Each value represents the median and interquartile range.

After one‐legged knee extension exercise, expression of CREB1‐target genes *NR4A3*,* MAFF*, and *SIK1* in exercised muscle increased by two and one order of magnitude in the untrained (*P* < 0.01) and trained (*P* < 0.01–0.05) state, respectively. In all cases but one, there was no increase in expression of these genes in nonexercised muscle (Fig. 5).

### Effect of acute one‐legged continuous knee extension exercise on signaling proteins

In the untrained state, ACC^Ser79/222^ phosphorylation, a marker of AMPK activity, and dephosphorylation of CRTC2 in the exercised muscle increased 2 min after termination of one‐legged knee extension exercise by ~600% (*P* < 0.01) and ~75% (*P* < 0.05), respectively. No postexercise changes in phosphorylation of p38^Thr180/Tyr182^, CaMKII^Thr286^, and CREB1^Ser133^ were observed in untrained exercised muscle. No postexercise changes in the phosphorylation status of these proteins were found in trained exercised muscle, untrained and trained nonexercised muscle (Table 1).

**Table 1:** Phosphorylation of exercise‐related signaling proteins and dephosphorylation of CRTC2 in exercised *m. vastus lateralis* prior to and at 2 min after, the one‐legged continuous knee extension exercise

	Untrained muscle		Endurance‐trained muscle	
	Pre	2 min	Pre	2 min
ACC^Ser79/222^	0.034 (0.007–0.089)	0.223** (0.148–0.366)	0.019 (0.009–0.049)	0.057 (0.012–0.120)
p38^Thr180/Tyr182^	0.034 (0.011–0.116)	0.025 (0.019–0.060)	0.032 (0.013–0.054)	0.012 (0.004–0.029)
CaMKII^Thr286^	0.168 (0.151–0.191)	0.183 (0.132–0.272)	0.247 (0.145–0.437)	0.281 (0.194–0.371)
CREB1^Ser133^	0.051 (0.010–0.133)	0.068 (0.029–0.123)	0.092 (0.041–0.193)	0.100 (0.072–0.122)
Dephospho‐CRTC2	0.005 (0.003–0.033)	0.014* (0.002–0.042)	0.015 (0.003–0.050)	0.020 (0.005–0.040)

No changes in phosphorylation of these proteins were found in nonexercised muscle (data not shown). *P < 0.05,**P < 0.01, compared with pre‐exercise, *n* = 9–10. Each value represents the median and interquartile range.

## Discussion

### Effect of training on muscle oxidative capacity and performance

The 8 week cycling training program increased the aerobic performance of the knee extensor muscles and increased expression of mitochondrial markers in the *m. vastus lateralis* (Fig. 2). Importantly, performing one‐legged knee extension exercise with the same relative intensity before and after the 8 week training program triggered similar physiological responses, as evaluated by catecholamine levels in the blood and subjective rating of perceived exertion (Fig. 3).

### Expression of the PPARGC1A gene in trained and untrained muscle

Expression of the *PPARGC1A* gene via the canonical promoter in untrained exercised and nonexercised muscles increased by ~100% after one‐legged continuous knee extension exercise (Fig. 4A and B), that is, expression from the canonical promoter may be regulated by systemic factors. This systemic effect may be related to stress hormones, metabolites and myokines secreted by exercised muscle (Hashimoto et al. 2007; Kitaoka et al. 2016; Ost et al. 2016). Interestingly, no postexercise changes in expression from the canonical promoter were observed in trained muscle (after the 8 week training program) (Fig. 4B). This may be related to a reduction in systemic stimuli or a decrease in the sensitivity of muscle. Postexercise expression of the *PPARGC1A* gene via the alternative promoter in untrained and trained exercised muscle increased by two orders of magnitude, while that in nonexercised muscle was negligible (Fig. 4C and D), that is, expression via the alternative promoter is regulated by intramuscular factors.

### Regulation of PPARGC1A gene expression via the alternative promoter

A human myoblast study showed that CREB1 plays an important role in regulating expression of the *PPARGC1A* via the alternative promoter (Yoshioka et al. 2009). In contrast to nonexercised muscle, acute exercise substantially increased expression of CREB1‐related genes (*NR4A3*,* MAFF*,* SIK1*) in both untrained and trained exercised skeletal muscle (Fig. 5). This finding confirms (indirectly) that acute exercise activates CREB1, and that this activation depends on intramuscular stimuli. Similar results were obtained in another study that investigated exercise‐induced changes in the transcriptome in untrained exercised and nonexercised human muscle; the authors suggest that the most significant set of target genes in the exercised leg is controlled by CREB1 (Catoire et al. 2012).

Many exercise‐associated kinases (PKA, AMPK, p38, and CaMKII) may phosphorylate CREB1, thereby increasing its transcriptional activity (Shaywitz and Greenberg 1999; Johannessen and Moens 2007; Thomson et al. 2008). Here, exercise‐induced expression in nonexercised muscle via the alternative promoter did not increase markedly, despite increased catecholamine levels in the blood, that is, the *β*2AR‐cAMP‐PKA pathway played no role in regulating *PPARGC1A* expression via the alternative promoter. This conclusion supports previous findings from a human study showing no significant correlation between *PPARGC1A* expression via the alternative promoter and phosphorylation of PKA substrates, a marker of PKA activity, after acute exercise (Popov et al. 2015b). Our data show that AMPK may be responsible for the exercise‐induced increase in *PPARGC1A* expression via the alternative promoter, at least in the untrained state (Table 1). However, previous work shows no association between exercise‐ and metformin‐induced activation of AMPK and *PPARGC1A* expression via the alternative promoter in human skeletal muscle (Popov et al. 2014, 2017; Silvennoinen et al. 2015). Moreover, we found no exercise‐induced changes in CREB1^Ser133^ phosphorylation under any experimental conditions. There are ambiguous data regarding CREB1^Ser133^ phosphorylation in human muscle immediately after acute aerobic exercise (Widegren et al. 1998, 2000; Egan et al. 2010; Popov et al. 2015b; Brandt et al. 2016). The lack of increased CREB1^Ser133^ phosphorylation immediately after exercise may be explained in several ways. CREB1 binds to DNA (and regulates transcription) as a homo‐ or heterodimer with the cAMP‐dependent transcription factors ATFs, cAMP‐responsive element modulator, CREB‐related transcription factors, and proteins of the activator protein (AP) 1 complex family (FOS, JUN and others) (Hai and Curran 1991; Newman and Keating 2003); therefore, expression of CREB1‐related genes may depend on phosphorylation of many other CREB1‐related transcriptional factors. For instance, an exercise‐induced and intensity‐dependent increase in ATF‐2^Thr71^ phosphorylation occurs in human skeletal muscle (Egan et al. 2010). On the other hand, the transcriptional activity of CREB1 may be regulated by CREB‐regulated transcription coactivators (CRTCs) (Screaton et al. 2004; Altarejos and Montminy 2011). Indeed, in human and rodent myoblasts, CRTCs regulate expression of *PPARGC1A* via the alternative (Yoshioka et al. 2009; Bruno et al. 2014) and canonical (Wu et al. 2006; Bruno et al. 2014; Rahnert et al. 2016) promoters through CREB1. Moreover, overexpression of CRTCs in mouse myoblasts induces mitochondrial gene expression and enhances mitochondrial respiration in PGC‐1*α*‐dependent manner (Wu et al. 2006). We did see an increase in the dephosphorylated (activated) CRTC2 in untrained exercised muscles after one‐legged continuous knee extension exercise. Calcium‐dependent serine‐threonine phosphatase calcineurin plays an important role in activation of CRTC2 via dephosphorylation at Ser^171^ (Screaton et al. 2004; Altarejos and Montminy 2011) and, therefore, may be responsible for the exercise‐induced activation of CRTC2 and CRTC2‐dependent *PPARGC1A* expression via the alternative promoter in untrained muscle. We did not find the exercise‐induced increase in the dephosphorylated CRTC2 after the 8 week training program (Table 1); this may partially explain the less pronounced increase in postexercise *PPARGC1A* expression via the alternative promoter (Fig. 4C).

### A possible role of transcriptional repressors in regulation of the alternative promoter

**Figure 6:**
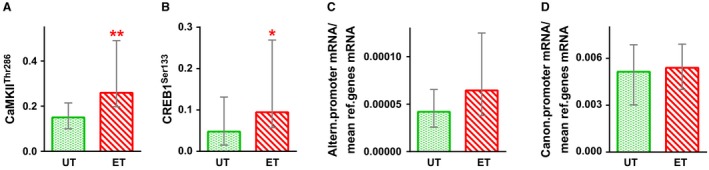
The basal phosphorylation of CaMKII^T^ ^hr286^ (A) and CREB1^Ser133^ (B), and the basal expression of *PPARCG1A* via the alternative (C) and proximal (D) promoters in untrained (UT, green bars) and endurance‐trained (ET, red bars) *m. vastus lateralis*. * *P* < 0.05 and ** *P* < 0.01 compared with untrained muscle; *n* = 9–10. The mean for both legs was calculated for each subject; each value represents the median and interquartile range.

**Figure 7:**
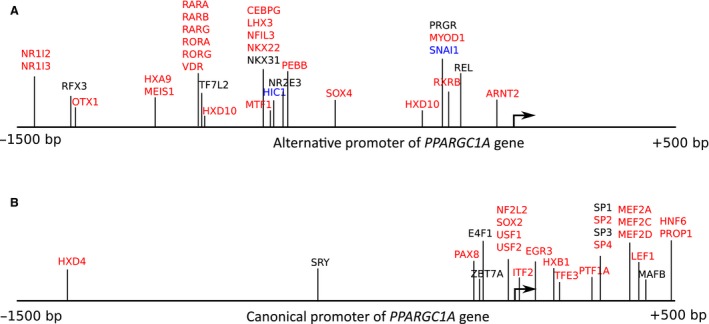
Unique transcription factors associated with the alternative (A) and canonical (B) promoter regions. The alternative and canonical promoter regions (−1500 – +500) of the human and mouse *PPARGC1A* gene were aligned. Next, conserved regulatory motifs and corresponding transcription factors were identified and transcription factors unique to each promoter were chosen. Unique transcription activators are indicated in *red*; transcription factors that may work as activators and repressors are indicated in *black*; and transcription repressors are indicated in *blue*.

According to previous human (Rose et al. 2007) and rodent (Feng et al. 2013) studies, we found the increase in the basal phosphorylation of CaMKII^Thr286^ and CREB1^Ser133^ in endurance‐trained muscle compared to untrained muscle. Surprisingly, the basal expression of *PPARCG1A* via the alternative promoter did not increase in trained muscle (Fig. 6). It is logical to assume that transcriptional repressors play a role in regulation of the alternative promoter: they are active at rest (and completely supress expression via the promoter), but may be deactivated by exercise‐related stimuli. We checked the possibility using a computational approach. The molecular mechanisms regulating *PPARGC1A* expression in muscle via the alternative promoter are conserved; both at rest and after exercise of differing intensity, mouse (Tadaishi et al. 2011b; Wen et al. 2014) and human (Popov et al. 2015b) skeletal muscles demonstrate the same promoter‐driven expression pattern. Bearing in mind that the canonical promoter shows constitutive exercise‐independent expression, we compared both promoter regions and chose unique conserved motifs within each. We identified several motifs associated with transcriptional activators and repressors (Fig. 7). In contrast to the canonical promoter, many unique regulatory motifs lie upstream of the transcription start site in the alternative promoter. Interestingly, unique motifs associated with transcription repressors were found in the alternative promoter region only (Zinc finger protein SNAI1 and Hypermethylated in cancer 1 protein HIC1). Both proteins, according to The Human Protein Atlas (http://www.proteinatlas.org/), are highly expressed in human skeletal muscle. Interestingly, SNAI1 was associated with a motif overlapping the MyoD‐related E‐box motif. A previous study shows that MyoD in human myoblasts may activate expression via the alternative promoter through the E‐box motif (Yoshioka et al. 2009). Therefore, exercise‐induced deactivation of SNAI1 may play an important role in activating gene expression via the alternative promoter in a MyoD‐dependent manner.

## Conclusions

Exercise‐induced expression of the *PPARGC1A* gene in untrained human muscle via the canonical promoter may be regulated by systemic factors, while that in trained muscle shows constitutive expression both at rest and after exercise. Exercise‐induced expression of the *PPARGC1A* gene via the alternative promoter is related to intramuscular factors, for instance, to activation of CRTC2‐CREB1 and may be regulated by repressors SNAI1 and HIC1, which are associated with the alternative promoter only. These results help us to better understand the molecular mechanism(s) underlying promoter‐specific regulation of the *PPARGC1A* gene expression in human skeletal muscle.

## Conflict of Interests

There are no conflict of interests.
